# Supplementary material for: Elevated temperature fatally disrupts nuclear divisions in the early Drosophila embryo
Source: Nat Commun. 2026 May 21;17:4601. doi: 10.1038/s41467-026-72982-9 (PMC13197460; doi:10.1038/s41467-026-72982-9)
Supplement: Supplementary file 1 — Supplementary information [file 41467_2026_72982_MOESM1_ESM.pdf]

Elevated temperature fatally disrupts nuclear divisions in the early *Drosophila* embryo.

Girish Kale[1,2,*], Pratika Agarwal[1], J Jaime Diaz-Larrosa[1], Steffen Lemke[1,2,*]

[1] Centre for Organismal Studies, University of Heidelberg, Heidelberg. Germany

[2] Institute of Biology, University of Hohenheim, Stuttgart, Germany

* Correspondence: G.K. (girish.kale@uni-hohenheim.de) and S.L. (steffen.lemke@uni-hohenheim.de)

Supplementary information

1) Summary of this file: Supplementary Information file contains Supplementary Figures, Supplementary Data legends, and Supplementary references associated with Supplementary Data.

2) Additionally, associated with this work is a ZIP file titled "Supplementary Data".

Summary of ZIP file titled "Supplementary Data": Supplementary Data file containing Excel files (.xlsx) associated with the analysis of clinal genes (Supplementary Data 1 and 2) and a list of genotypes associated with various figure panels (Supplementary Data 3).

Supplementary Information file

Table of Contents

Supplementary Figures: 2

Supplementary Data legends: 9

Supplementary References: 10

Supplementary Figures:

Figure S1: Blastoderm holes appear in a fraction of embryos at elevated temperature.

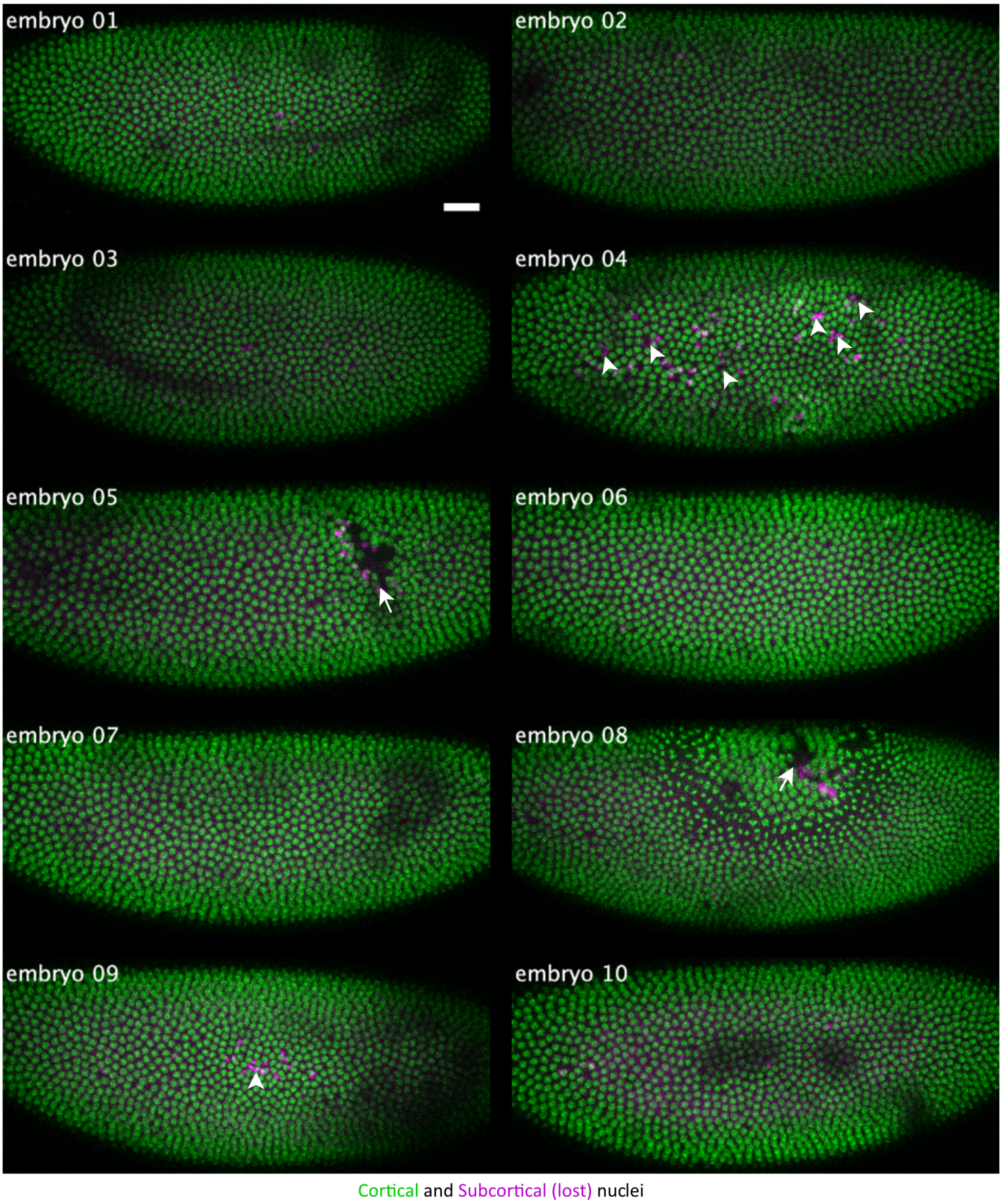

The figure shows the snapshots from live embryos, expressing Histone-RFP, from early interphase 14, in embryos at elevated temperature (29°C). The nuclei are labelled as a function of their distance from the embryo surface. Green, cortical nuclei; magenta, sub-cortical nuclei (see also schematic in Figure 4B). Arrows, blastoderm holes; arrowheads, small gaps in blastoderm. For embryos with blastoderm holes (embryos 05, and 08), we see many nuclei in magenta. In contrast, in the case of embryos with small gaps (embryos 04, and 09), we can see only a few nuclei in magenta.

Scale bar, 25 µm.

Figure S2: Defective nuclei take different amounts of time to be expelled from the embryo cortex.

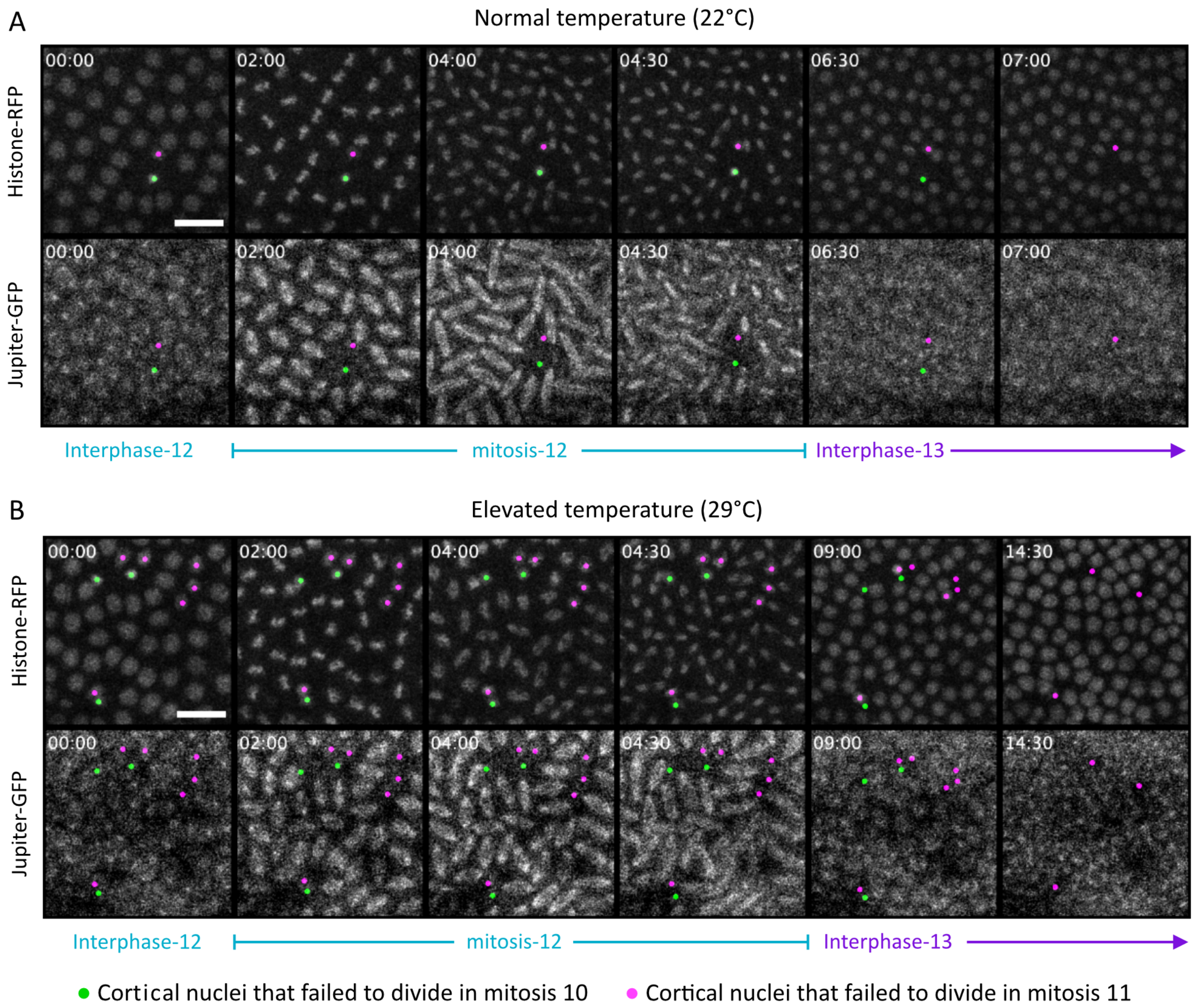

(A, B) Montages from live embryos, with a few representative nuclei tracked from late interphase 12 to interphase 13. Top panels, Histone-RFP signal marking nuclei; bottom panels, Jupiter-GFP signal marking microtubules. Nuclei tagged in green/magenta are the ones that arise from mitotic defects during mitosis 10/11. All of these are expelled during interphase 13. Notice the lack of spindle formation around the tagged nuclei. Also, note that the nuclei expelled by the 5th timepoint are not shown in the 6th timepoint. (A) An embryo developing at normal temperature (22°C), where 1 magenta and 1 green nuclei are followed. (B) An embryo developing at elevated temperature (29°C), where 6 magenta and 3 green nuclei are followed.

Scale bars, 20 µm. Insets, time in min:sec.

Figure S3: Nuclear movement along surface-yolk axis is amplified at elevated temperature.

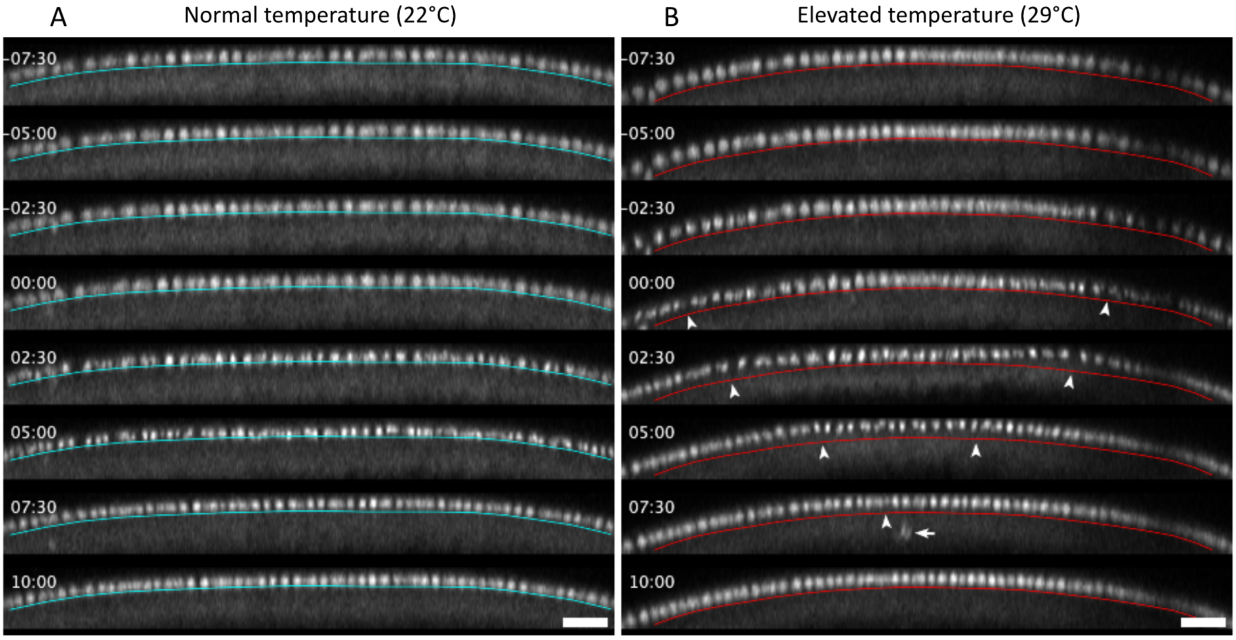

Cross-sections from live embryos, expressing Histone-RFP, from late interphase 13 till early interphase 14, in embryos developing either at normal temperature (22°C, A) or at elevated temperature (29°C, B). Colored lines, basal tip of nuclei in the first frame; insets, time since initiation of anaphase 13 at the left/right extreme of the images (which is also the first time we see the shift from baseline in embryo at elevated temperature); arrowheads, local peak of the nuclear shift from the baseline; arrow, expelled nuclei.

Scale bars, 25 µm. Cross-sections are in the lateral region of the embryos, anterior to the left.

Figure S4: Nuclear cycle asynchrony at elevated temperature during embryo development.

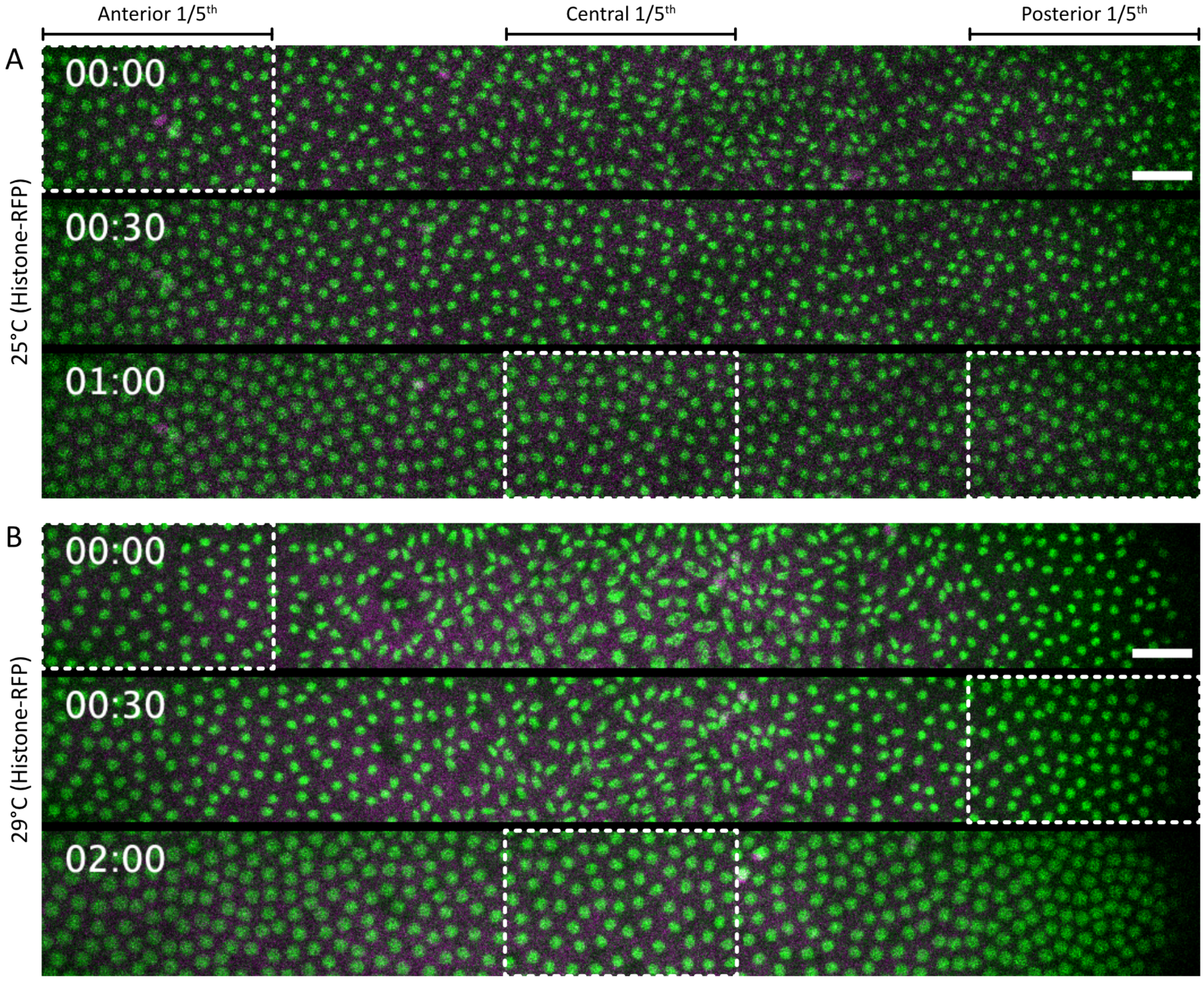

Cortical and Subcortical (lost) nuclei

To assess the asynchrony in nuclear cycles, we identified the phases of the nuclear cycle along the AP axis. (A, B) Montages show the transition from nuclear cycle 13 to early interphase 14, from live embryos at 25°C (A), and at 29°C (B). Histone-RFP marks the nuclei: green, cortical nuclei; magenta, subcortical (lost) nuclei (see also schematic in Figure 4B); inset, time in min:sec. The anterior, central, and posterior 1/5th of the field of view are as indicated. Time 00:00 is when the anterior 1/5th of the embryo enters interphase 14. Boxes highlight entry into interphase 14 in that region.

Scale bars, 20 µm.

Figure S5: Embryonic lethality and mitotic failures at elevated temperature in *baz* or *dlg* overexpressions.

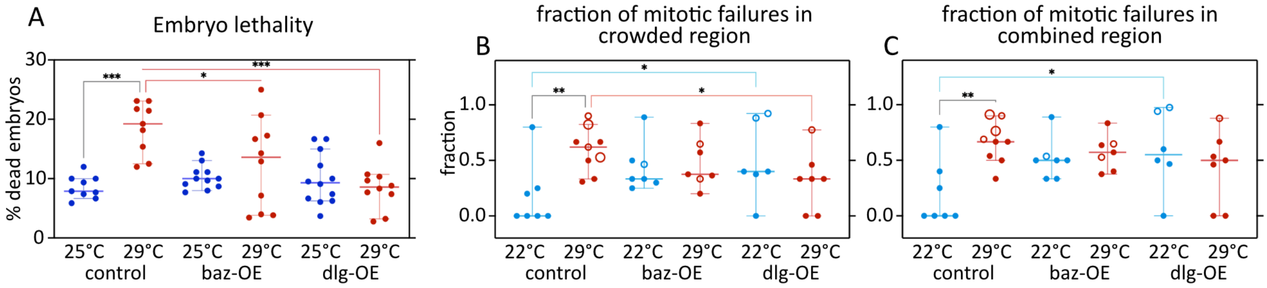

(A) Scatter plot shows distribution of percent lethality in embryos from various genotypes, at normal (25°C) vs elevated temperature (29°C), calculated based on the fraction of embryos that don't hatch into larvae. n= 9 (25°C control), 9 (29°C control), 11 (25°C baz-OE), 10 (29°C baz-OE), 12 (25°C dlg-OE), 10 (29°C dlg-OE) samples; each sample has ≥12 embryos.

(B, C) Scatter plots show distribution of the fraction of mitotic failures in crowded regions (B), and in combined regions (C), in the embryos at normal (22°C) vs elevated temperature (29°C). n= 7 (22°C control), 9 (29°C control), 7 (22°C baz-OE), 7 (29°C baz-OE), 6 (22°C dlg-OE), 7 (29°C dlg-OE) embryos.

Plots show scatters with median, whiskers show 95% CI. In B and C, large hollow markers, embryos with blastoderm holes; small hollow markers, embryos with small gaps in the blastoderm. Non-parametric Kruskal-Wallis test with Dunn's uncorrected test for multiple comparisons: *, $p<0.05$; **, $p<0.01$; ***, $p<0.001$; non-significant comparisons are not shown. Source data and exact p-values for plots are available in the Source Data file.

Figure S6: Elevated temperature disrupts the association between nuclei and F-actin caps.

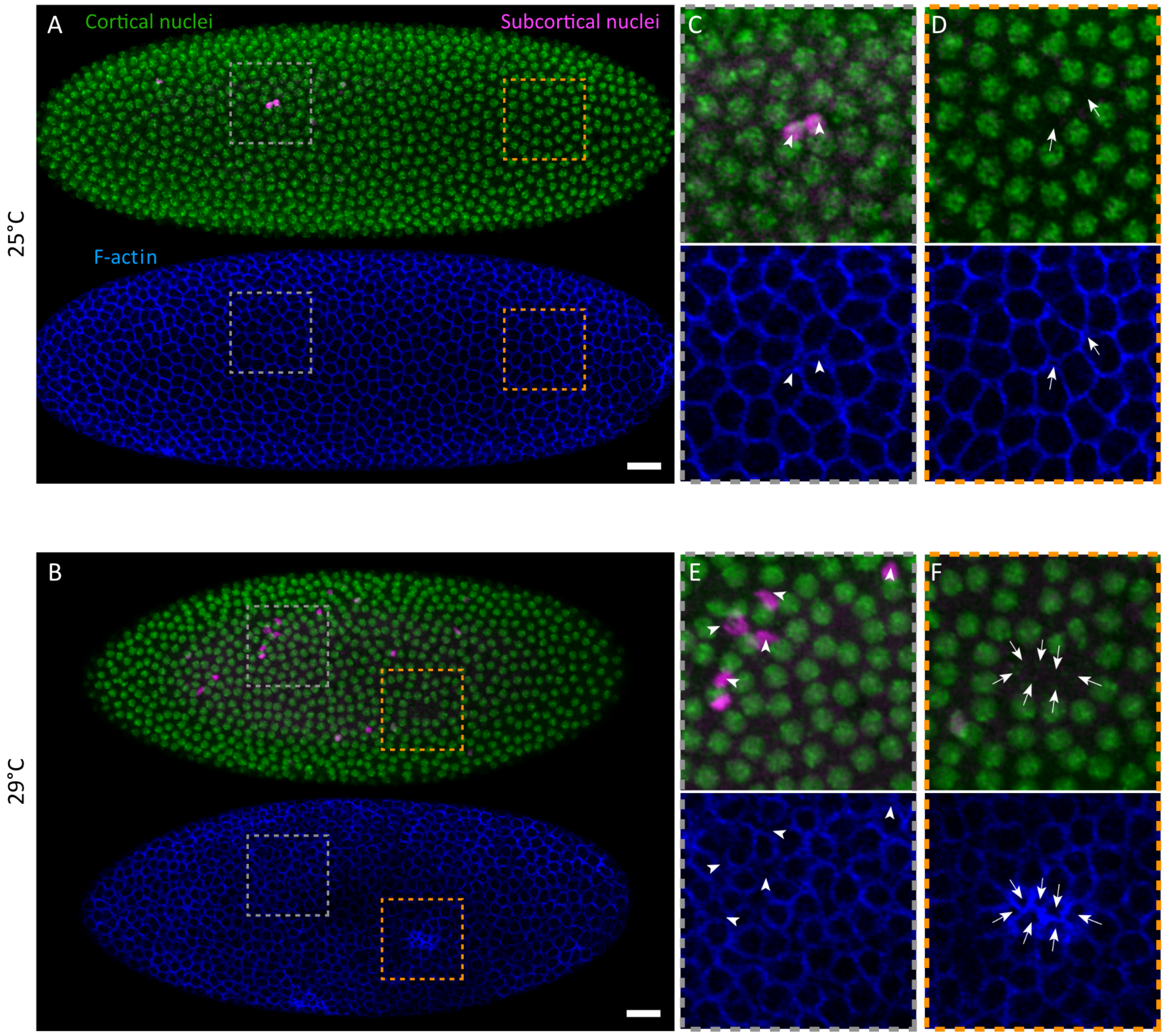

Distribution of nuclei and F-actin in embryos during interphase 13, in fixed embryos that were developing either at normal temperatures (A, C, D), or elevated temperature (B, E, F). (A, B) The images show SUM projections of the DAPI-stained nuclei (top image) and Phallacidin-stained F-actin (bottom image) of representative embryos. In the top images, the nuclei are labelled as a function of their distance from the embryo surface, where green indicates cortical, and magenta indicates sub-cortical nuclei (see also schematic in Figure 2B). The yolk nuclei are not shown. Anterior is on the left, dorsal up. (C-F) The top and bottom images show a zoomed-in view of the indicated regions in panels A and B. Arrowheads, expelled nuclei (C, E top panels) and corresponding gaps between the actin-caps (C, E bottom panels); arrows, gaps between neighbouring nuclei (D, F top panels) and corresponding gaps between the actin-caps at normal temperature (D, bottom panel) and “empty actin caps” at elevated temperature (F, bottom panel).

Scale bars, 25µm. The boxed regions are 60x60 µm.

Figure S7: *mud* or *insc* overexpression does not ameliorate embryo survival at elevated temperature.

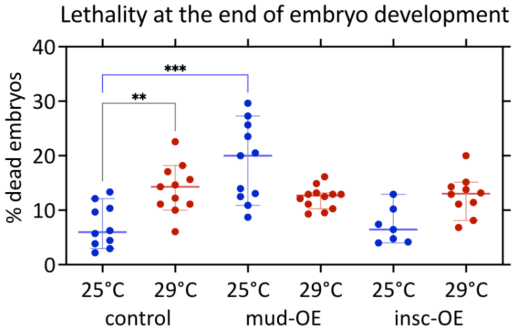

Scatter plot shows the distribution of percent lethality in embryos from various genotypes, at normal (25°C) vs elevated temperature (29°C), calculated based on the fraction of embryos that don't hatch into larvae. n= 10 (22°C control), 11 (29°C control), 11 (22°C mud-OE), 12 (29°C mud-OE), 7 (22°C insc-OE), 10 (29°C insc-OE) samples; each sample has ≥21 embryos. As we can see in the plot, neither *mud* nor *insc* overexpression rescues the embryo lethality at elevated temperature, with *mud* overexpression increasing embryo lethality even at normal temperature (25°C).

The plot shows scatters with a median, while whiskers show 95% CI. Non-parametric Kruskal-Wallis test with Dunn's uncorrected test for multiple comparisons: *, p<0.05; **, p<0.01; ***, p<0.001; non-significant differences are omitted. Source data and exact p-values for the plot are available in the Source Data file.

Supplementary Data legends:

Supplementary Data 1: This table contains genes that are identified as being clinal in different continents. Columns A to C contain lists of studies, and under the studies is a list of genes that are identified in these studies as being clinal. Columns A, B, and C respectively list studies that have data from the continents of Australia and/or Australasia (AUS) [1–10], North and/or Central America (USA) [1,8,18–20,10–17], and Europe and/or North Africa (EU) [1,7,21,22]. We collect all the genes associated with 'spindle dynamics' (Figure 10D), to compile a list of potential candidate genes in Column F. For each gene in column F, we count the number of times that gene appears in columns A to C, counting a maximum of once per column, to respectively populate columns G to I. For each gene in column F, column J contains the sum of values in G, H, and I, to indicate the number of continents in which a gene is clinal. Each gene gets a score from 0 to 3, and we consider the gene to be a relevant candidate if it is found in at least 2 columns, i.e., with a score of 2 or 3.

We collect all the genes associated with the GO term 'metabolic process', to compile a control list of candidate genes in Column L. For each gene in column L, we count the number of times that gene appears in columns A to C, counting a maximum of once per column, to respectively populate columns M to O. For each gene in column L, column P contains the sum of values in M, N, and O, to indicate the number of continents in which a gene is clinal. Thus, each gene gets a score from 0 to 3.

Supplementary Data 2: Column A lists candidates from Supplementary Data 1 column F that have a score of 2 or higher, i.e., those that show significant selectable variation in natural populations. For these genes, we query FlyBase (http://flybase.org/batchdownload) for additional details like gene name (column B), gene symbol (column C), and the level of expression during the first 2 hours of embryo development (column D). The level of expression is based on published transcriptomics data[23], and would correspond to a maternal pool of mRNAs, which is the most relevant mRNA source for this study. The candidate genes clinal in all 3 continents are highlighted in green. Columns F-J present a table with counts of genes that are associated with various categories of genes in Supplementary Data 1 columns F and L, and a further breakdown of how many of these genes are clinal in how many continents. The breakdown is also plotted as pie charts in Figure 10E.

Supplementary Data 3: This table lists various figure panels (columns A and B), and the related parental genotypes, i.e., the genotypes of females (column C) and males (column D) present in the embryo collection cages for the experiment related to a figure panel.

Supplementary References:

1. Oakeshott, J.G., McKechnie, S.W., and Chambers, G.K. (1984). Population genetics of the metabolically related Adh, Gpdh and Tpi polymorphisms in Drosophila melanogaster I. Geographic variation in Gpdh and Tpi allele frequencies in different continents. Genetica *63*, 21–29. 10.1007/BF00137461.
2. McColl, G., and McKechnie, S.W. (1999). The Drosophila heat shock hsr-omega gene: An allele frequency cline detected by quantitative PCR. Mol. Biol. Evol. *16*, 1568–1574. 10.1093/oxfordjournals.molbev.a026069.
3. Bettencourt, B.R., Kim, I., Hoffmann, A.A., and Feder, M.E. (2002). Response to natural and laboratory selection at the Drosophila hsp70 genes. Evolution (N. Y). *56*, 1796–1801. 10.1111/j.0014-3820.2002.tb00193.x.
4. Frydenberg, J., Hoffmann, A.A., and Loeschcke, V. (2003). DNA sequence variation and latitudinal associations in hsp23, hsp26 and hsp27 from natural populations of Drosophila melanogaster. Mol. Ecol. *12*, 2025–2032. 10.1046/j.1365-294X.2002.01882.x.
5. Umina, P.A., Weeks, A.R., Kearney, M.R., McKechnie, S.W., and Hoffmann, A.A. (2005). Evolution: A rapid shift in a classic clinal pattern in Drosophila reflecting climate change. Science (80-.). *308*, 691–693. 10.1126/science.1109523.
6. Sawyer, L.A., Sandrelli, F., Pasetto, C., Peixoto, A.A., Rosato, E., Costa, R., and Kyriacou, C.P. (2006). The period gene Thr-Gly polymorphism in Australian and African Drosophila melanogaster populations: Implications for selection. Genetics *174*, 465–480. 10.1534/genetics.106.058792.
7. Weeks, A.R., Mckechnie, S.W., and Hoffmann, A.A. (2006). In search of clinal variation in the period and clock timing genes in Australian Drosophila melanogaster populations. J. Evol. Biol. *19*, 551–557. 10.1111/j.1420-9101.2005.01013.x.
8. Rand, D.M., Weinreich, D.M., Lerman, D., Folk, D., and Gilchrist, G.W. (2010). Three selections are better than one: Clinal variation of thermal QTL from independent selection experiments in drosophila. Evolution (N. Y). *64*, 2921–2934. 10.1111/j.1558-5646.2010.01039.x.
9. Kolaczkowski, B., Kern, A.D., Holloway, A.K., and Begun, D.J. (2011). Genomic differentiation between temperate and tropical Australian populations of Drosophila melanogaster. Genetics *187*, 245–260. 10.1534/genetics.110.123059.
10. Paaby, A.B., Bergland, A.O., Behrman, E.L., and Schmidt, P.S. (2014). A highly pleiotropic amino acid polymorphism in the Drosophila insulin receptor contributes to life-history adaptation. Evolution *68*, 3395–3409. 10.1111/evo.12546.
11. Vigue, C.L., and Johnson, F.M. (1973). Isozyme Variability in Species of the Genus Drosophila. VI. Frequency-Property-Environment Relationships of Allelic Alcohol Dehydrogenases in D. melanogaster. Biochem. Genet. *9*, 213–227.
12. Berry, A., and Kreitman, M. (1993). Molecular analysis of an allozyme cline: alcohol dehydrogenase in Drosophila melanogaster on the east coast of North America. Genetics *134*, 869–893. 10.1093/genetics/134.3.869.
13. Duvernell, D.D., and Eanes, W.F. (2000). Contrasting molecular population genetics of four hexokinases in Drosophila melanogaster, D. simulans and D. yakuba. Genetics *156*, 1191–1201. 10.1093/genetics/156.3.1191.
14. Schmidt, P.S., Duvernell, D.D., and Eanes, W.F. (2000). Adaptive evolution of a candidate gene for aging in Drosophila. Proc. Natl. Acad. Sci. *97*, 10861–10865. 10.1073/pnas.190338897.

15. Verrelli, B.C., and Eanes, W.F. (2001). Clinal variation for amino acid polymorphisms at the Pgm locus in Drosophila melanogaster. Genetics *157*, 1649–1663. 10.1093/genetics/157.4.1649.

16. Duvernell, D.D., Schmidt, P.S., and Eanes, W.F. (2003). Clines and adaptive evolution in the methuselah gene region in Drosophila melanogaster. Mol. Ecol. *12*, 1277–1285. 10.1046/j.1365-294X.2003.01841.x.

17. Sezgin, E., Duvernell, D.D., Matzkin, L.M., Duan, Y., Zhu, C.T., Verrelli, B.C., and Eanes, W.F. (2004). Single-locus latitudinal clines and their relationship to temperate adaptation in metabolic genes and derived alleles in Drosophila melanogaster. Genetics *168*, 923–931. 10.1534/genetics.104.027649.

18. Schmidt, P.S., and Paaby, A.B. (2008). Reproductive diapause and life-history clines in North American populations of Drosophila melanogaster. Evolution (N. Y). *62*, 1204–1215. 10.1111/j.1558-5646.2008.00351.x.

19. Fabian, D.K., Kapun, M., Nolte, V., Kofler, R., Schmidt, P.S., Schlötterer, C., and Flatt, T. (2012). Genome-wide patterns of latitudinal differentiation among populations of Drosophila melanogaster from North America. Mol. Ecol. *21*, 4748–4769. 10.1111/j.1365-294X.2012.05731.x.

20. Machado, H.E., Bergland, A.O., O'Brien, K.R., Behrman, E.L., Schmidt, P.S., and Petrov, D.A. (2016). Comparative population genomics of latitudinal variation in Drosophila simulans and Drosophila melanogaster. Mol. Ecol. *25*, 723–740. 10.1111/mec.13446.

21. Costa, R., Peixoto, A.A., Barbujani, G., and Kyriacou, C.P. (1992). A latitudinal cline in a Drosophila clock gene. Proc. R. Soc. London, 43–49.

22. Kapun, M., Barrón, M.G., Staubach, F., Obbard, D.J., Wiberg, R.A.W., Vieira, J., Goubert, C., Rota-Stabelli, O., Kankare, M., Bogaerts-Márquez, M., et al. (2020). Genomic Analysis of European Drosophila melanogaster Populations Reveals Longitudinal Structure, Continent-Wide Selection, and Previously Unknown DNA Viruses. Mol. Biol. Evol. *37*, 2661–2678. 10.1093/molbev/msaa120.

23. Graveley, B.R., Brooks, A.N., Carlson, J.W., Duff, M.O., Landolin, J.M., Yang, L., Artieri, C.G., van Baren, M.J., Boley, N., Booth, B.W., et al. (2011). The developmental transcriptome of Drosophila melanogaster. Nature *471*, 473–479. 10.1038/nature09715.
