# Supplementary material for: Elevated temperature fatally disrupts nuclear divisions in the early Drosophila embryo
Source: Nat Commun. 2026 May 21;17:4601. doi: 10.1038/s41467-026-72982-9 (PMC13197460; doi:10.1038/s41467-026-72982-9)
Supplement: Supplementary file 2 — Description of Additional Supplementary Files [file 41467_2026_72982_MOESM2_ESM.pdf]

Title: Supplementary Data 1

Description: This table contains genes that are identified as being clinal in different continents. Columns A to C contain lists of studies, and under the studies is a list of genes that are identified in these studies as being clinal. Columns A, B, and C respectively list studies that have data from the continents of Australia and/or Australasia (AUS) 1–10, North and/or Central America (USA) 1,8,18–20,10–17, and Europe and/or North Africa (EU) 1,7,21,22. We collect all the genes associated with 'spindle dynamics' (Figure 10D), to compile a list of potential candidate genes in Column F. For each gene in column F, we count the number of times that gene appears in columns A to C, counting a maximum of once per column, to respectively populate columns G to I. For each gene in column F, column J contains the sum of values in G, H, and I, to indicate the number of continents in which a gene is clinal. Each gene gets a score from 0 to 3, and we consider the gene to be a relevant candidate if it is found in at least 2 columns, i.e., with a score of 2 or 3. We collect all the genes associated with the GO term 'metabolic process', to compile a control list of candidate genes in Column L. For each gene in column L, we count the number of times that gene appears in columns A to C, counting a maximum of once per column, to respectively populate columns M to O. For each gene in column L, column P contains the sum of values in M, N, and O, to indicate the number of continents in which a gene is clinal. Thus, each gene gets a score from 0 to 3.

Title: Supplementary Data 2

Description: Column A lists candidates from Supplementary Data 1 column F that have a score of 2 or higher, i.e., those that show significant selectable varia on in natural populations. For these genes, we query FlyBase (http://flybase.org/batchdownload) for additional details like gene name (column B), gene symbol (column C), and the level of expression during the first 2 hours of embryo development (column D). The level of expression is based on published transcriptomics data23, and would correspond to a maternal pool of mRNAs, which is the most relevant mRNA source for this study. The candidate genes clinal in all 3 continents are highlighted in green. Columns F-J present a table with counts of genes that are associated with various categories of genes in Supplementary Data 1 columns F and L, and a further breakdown of how many of these genes are clinal in how many continents. The breakdown is also plotted as pie charts in Figure 10E.

Title: Supplementary Data 3

Description: This table lists various figure panels (columns A and B), and the related parental genotypes, i.e., the genotypes of females (column C) and males (column D) present in the embryo collection cages for the experiment related to a figure panel.
