# Supplementary material for: Elevated temperature fatally disrupts nuclear divisions in the early Drosophila embryo
Source: Nat Commun. 2026 May 21;17:4601. doi: 10.1038/s41467-026-72982-9 (PMC13197460; doi:10.1038/s41467-026-72982-9)
Supplement: Supplementary file 4 — Reporting Summary [file 41467_2026_72982_MOESM4_ESM.pdf]

nature portfolio

Corresponding author(s): Girish Kale, Steffen Lemke

Last updated by author(s): Apr 13, 2026

Reporting Summary

Nature Portfolio wishes to improve the reproducibility of the work that we publish. This form provides structure for consistency and transparency in reporting. For further information on Nature Portfolio policies, see our Editorial Policies and the Editorial Policy Checklist.

Statistics

For all statistical analyses, confirm that the following items are present in the figure legend, table legend, main text, or Methods section.

n/a	Confirmed	
☐	☒	The exact sample size (*n*) for each experimental group/condition, given as a discrete number and unit of measurement
☒	☐	A statement on whether measurements were taken from distinct samples or whether the same sample was measured repeatedly
☐	☒	The statistical test(s) used AND whether they are one- or two-sided<br>*Only common tests should be described solely by name; describe more complex techniques in the Methods section.*
☒	☐	A description of all covariates tested
☒	☐	A description of any assumptions or corrections, such as tests of normality and adjustment for multiple comparisons
☐	☒	A full description of the statistical parameters including central tendency (e.g. means) or other basic estimates (e.g. regression coefficient) AND variation (e.g. standard deviation) or associated estimates of uncertainty (e.g. confidence intervals)
☐	☒	For null hypothesis testing, the test statistic (e.g. *F*, *t*, *r*) with confidence intervals, effect sizes, degrees of freedom and *P* value noted<br>*Give P values as exact values whenever suitable.*
☒	☐	For Bayesian analysis, information on the choice of priors and Markov chain Monte Carlo settings
☒	☐	For hierarchical and complex designs, identification of the appropriate level for tests and full reporting of outcomes
☒	☐	Estimates of effect sizes (e.g. Cohen's *d*, Pearson's *r*), indicating how they were calculated

Our web collection on statistics for biologists contains articles on many of the points above.

Software and code

Policy information about availability of computer code

Data collection	This study uses proprietary software to control the microscope hardware during image acquisition (data collection). However, the softwares themselves has no bearing on the results.
Data analysis	For data analysis, this study uses custom-made ImageJ/FIJI (Version 2.16.0/1.54p) macros, Microsoft Excel (Version 16) and GraphpadPrism (Version 9) for data analysis; the macros and scripts used to facilitate data processing are available on request. Other software used was Affinity Designer (2.6.3).

For manuscripts utilizing custom algorithms or software that are central to the research but not yet described in published literature, software must be made available to editors and reviewers. We strongly encourage code deposition in a community repository (e.g. GitHub). See the Nature Portfolio guidelines for submitting code & software for further information.

Data

Policy information about availability of data

All manuscripts must include a data availability statement. This statement should provide the following information, where applicable:

- Accession codes, unique identifiers, or web links for publicly available datasets
- A description of any restrictions on data availability
- For clinical datasets or third party data, please ensure that the statement adheres to our policy

Authors confirm that Source Data are provided with this paper. Any associated raw data are available upon request from corresponding authors.

Research involving human participants, their data, or biological material

Policy information about studies with human participants or human data. See also policy information about sex, gender (identity/presentation), and sexual orientation and race, ethnicity and racism.

Reporting on sex and gender	N/A
Reporting on race, ethnicity, or other socially relevant groupings	N/A
Population characteristics	N/A
Recruitment	N/A
Ethics oversight	N/A

Note that full information on the approval of the study protocol must also be provided in the manuscript.

Field-specific reporting

Please select the one below that is the best fit for your research. If you are not sure, read the appropriate sections before making your selection.

[x] Life sciences [ ] Behavioural & social sciences [ ] Ecological, evolutionary & environmental sciences

For a reference copy of the document with all sections, see nature.com/documents/nr-reporting-summary-flat.pdf

Life sciences study design

All studies must disclose on these points even when the disclosure is negative.

Sample size	No statistical method was used to predetermine sample size. The sample size was based on previous studies in the field and indicated in the Figure or Figure legends.
Data exclusions	No datasets were excluded from the analysis; all data resulting from valid experiments were included. Valid experiments were defined as experiments that were free of technical, instrumental, reagent and sample-related issues.
Replication	All experiments were highly reproducible, including those showing multiple classes of phenotypes under given experimental manipulations. Consistency across replicates is captured in each experiment by the statistics reported and reflected in the statistic tests performed.
Randomization	Sample randomization was not possible for experiments in which temperature perturbations were introduced, as all individuals of the given genotype were under such a perturbation.
Blinding	Blinding was not performed during data collection. It was infeasible in most experiments as knowledge of the experimental perturbations was either required for their execution or was a part of the data collection procedure. Blinding was not performed during data analysis as results typically were qualitatively or quantitatively distinct between experimental groups, or was unnecessary in cases where automatic imaging analysis methods were used, or infeasible when features of the phenotypes were apparent to the investigators which of the perturbations had been administered to the samples. Detailed methods and criteria for data analysis were extensively described in Methods and Supplementary Methods to ensure objectivity and reproducibility of results.

Reporting for specific materials, systems and methods

We require information from authors about some types of materials, experimental systems and methods used in many studies. Here, indicate whether each material, system or method listed is relevant to your study. If you are not sure if a list item applies to your research, read the appropriate section before selecting a response.

Materials & experimental systems

n/a	Involved in the study	
x		Antibodies
x		Eukaryotic cell lines
x		Palaeontology and archaeology
	x	Animals and other organisms
x		Clinical data
x		Dual use research of concern
x		Plants

Methods

n/a	Involved in the study	
x		ChIP-seq
x		Flow cytometry
x		MRI-based neuroimaging

Animals and other research organisms

Policy information about studies involving animals; ARRIVE guidelines recommended for reporting animal research, and Sex and Gender in Research

Laboratory animals	Embryos of Drosophila melanogaster were used in this study and all experiments were carried out according to the equivalent of Campos-Ortega staging for D. melangoaster between stage 0 and larval hatching.
Wild animals	The study did not involve wild animals.
Reporting on sex	The experimental procedure did not allow identifying the sex of the embryos at the time of the experiment.
Field-collected samples	The study did not involve samples collected from the wild.
Ethics oversight	No ethical approval or guidance was required for this study of invertebrate developmental processes.

Note that full information on the approval of the study protocol must also be provided in the manuscript.

Plants

Seed stocks	N/A
Novel plant genotypes	N/A
Authentication	N/A
